# Environmental-Driven Approach towards Level 5 Self-Driving

**DOI:** 10.3390/s24020485

**Published:** 2024-01-12

**Authors:** Mohammad Hurair, Jaeil Ju, Junghee Han

**Affiliations:** School of Electronics and Information Engineering, Korea Aerospace University, 76 Hanggongdaehang-ro, Goyang-si 412-791, Gyeonggi-do, Republic of Korea; hurair@kau.ac.kr (M.H.); 2020124223@kau.kr (J.J.)

**Keywords:** autonomous driving, level 5, realtime, end-to-end delay, machine learning

## Abstract

As technology advances in almost all areas of life, many companies and researchers are working to develop fully autonomous vehicles. Such level 5 autonomous driving, unlike levels 0 to 4, is a driverless vehicle stage and so the leap from level 4 to level 5 autonomous driving requires much more research and experimentation. For autonomous vehicles to safely drive in complex environments, autonomous cars should ensure end-to-end delay deadlines of sensor systems and car-controlling algorithms including machine learning modules, which are known to be very computationally intensive. To address this issue, we propose a new framework, i.e., an environment-driven approach for autonomous cars. Specifically, we identify environmental factors that we cannot control at all, and controllable internal factors such as sensing frequency, image resolution, prediction rate, car speed, and so on. Then, we design an admission control module that allows us to control internal factors such as image resolution and detection period to determine whether given parameters are acceptable or not for supporting end-to-end deadlines in the current environmental scenario while maintaining the accuracy of autonomous driving. The proposed framework has been verified with an RC car and a simulator.

## 1. Introduction

Recently, the development of self-driving cars has emerged as a big issue. As technology advances in almost all areas of life, many companies are working to develop fully autonomous vehicles. The autonomous driving stage is specified by the Society of Autonomous Engineers (SAE) on six different levels [1]. Level 5 autonomous driving, unlike levels 0 to 4, is a driverless vehicle stage that does not require a driver. When the passenger specifies the destination, the system determines the destination and drives on its own without human intervention [2]. Control devices, such as the driver’s seat, accelerator, brake, and steering wheel, are not used. Many researchers [3,4,5] and companies [6,7,8,9] have made efforts to achieve level 5 autonomous driving, which intuitively responds to situations in a similar way to humans, even in situations that are encountered for the first time, based on information from various sensors. However, unfortunately, compared to the speed and difficulty of development from level 0 to level 4 autonomous driving, the leap from level 4 to level 5 autonomous driving requires much more research and experimentation. A “last mile problem” occurs when working towards level 5 self-driving.

**Figure 1:**
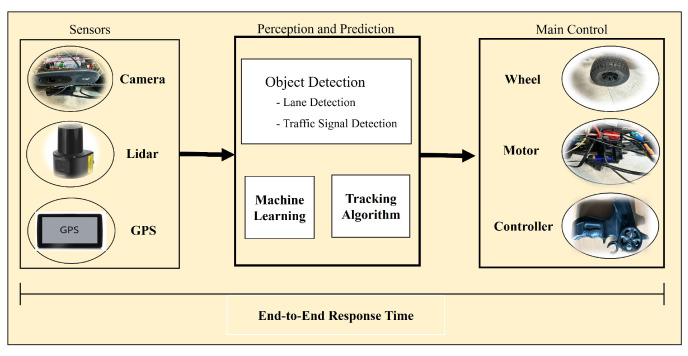
Architecture and processing flow of autonomous driving operation.

So far, most researchers have focused on improving the accuracy of perception and prediction methods and models. In contrast, there have been fewer studies on identifying the relationship and trade-off between runtime-accuracy and the end-to-end delay of autonomous driving operation [10,11]. Since autonomous driving operation consists of several different stages of process, as illustrated in Figure 1, it is very important to complete the flow of these processes within some time constraints. In particular, autonomous cars drive across a variety of environments such as through different weather, town traffic, and road conditions, and so on. To safely drive in these dynamic and complex environments, many computationally intensive software and sensors are involved. In this situation, it is very crucial to bound an end-to-end delay to deadlines with these complex flows of autonomous driving operations. From this perspective, this paper focuses on last mile problems that hinder level 5 autonomous driving and attempt to guide solutions to the identified limitations of current autonomous driving research.

The main contributions of the proposed paper can be summarized as follows:

- We investigated and discovered realtime factors that hinder level 5 autonomous driving. We identified environmental and internal factors with extensive experiments. We examined the detailed effects of these factors and measured the relationships and trade-offs among these factors based on experimental results, using both RC cars that we built and CARLA simulators;
- We proposed an environment-driven solution to address these issues. With the proposed solution, we set up internal factors (sensing frequency, image resolution, prediction rate, car speed, and so on) by considering the trade-off between runtime-accuracy and delay.

The rest of the paper is organized as follows. Section 2 introduces existing self-driving algorithms and discusses the problems with and limitations to real-world autonomous driving. Section 3 analyzes the trade-off among main environmental and internal factors to hinder autonomous driving and proposes a solution. In Section 4, we describe the experimental procedure and analyze the results. Finally, Section 5 wraps up this paper with a discussion.

## 2. Background

### 2.1. Level 5 Autonomous Driving

Many researchers and companies have made some assumptions and expectations that an early L4 prototype would be available by 2022 [3,4,5]. However, currently, the driver still must keep their hands on the wheel and turn it or spin a dial every few seconds to prove they are paying attention [6,7,8,9]. In reality, there are very few level 4 vehicles released as of 2023. This is because the development of the level 4 autonomous vehicle has been a complex and gradual process; several factors have contributed to the slower rollout of level 4 prototypes. Furthermore, level 5 cars are to be considered fully autonomous cars that can be driven without the driver paying attention. For this, we need to understand that no sensor is fully perfect; so, to avoid problems, we would need to use a variety of sensors so that the car could see the environment around them. It has been estimated that level 4 vehicles would have around 60 sensors. These sensors would include radar, LiDAR, cameras, GPS, etc. Some of them would be doing redundant work, but it would be good for safety and reliability. But to handle this many sensors, we need a specific computational power that works and delivers the results as soon as possible. Overall, to safely drive in the real-world, a level 5 car should support accurate results that are on time and not too late within the deadline by performing complex algorithms with these sensors and motors. To satisfy these requirements, we need to consider realtime issues as well as develop a perception and prediction mechanism. To easily illustrate the main idea, we simplify the target auto-driving car model as shown in Figure 1 (the proposed framework can be extended to accommodate more complex platforms of current commercial vehicles). As shown in this figure, autonomous driving operations consist of several different processing stages interconnected with each other. Hence, bounding end-to-end response delay to a threshold is not simple and straightforward; this is the main issue this paper attempts to address.

### 2.2. ML-Based Autonomous Driving Algorithms

Various machine learning algorithms (ML algorithms) are used by autonomous vehicles for different purposes like object detection, lane detection, steering angle prediction, etc., including  [12,13,14,15,16,17,18]. Gu et al. proposed an LSTM-based autonomous driving model [12]. Long Short-Term Memory (LSTM) network architecture is well suited for sequence prediction problems. LSTMs are designed to handle long-range dependencies in sequences, which is useful for tasks like trajectory prediction where the future states depend on multiple past states [13]. However, it is known to be hard for LSTMs to analyze the complex surrounding environment, and so an inherently LSTM-based model could lead to the accumulation of loss in real-world applications [12].

To extend the LSTM-based model’s applicability to autonomous car models, several modified models have been proposed. The DRL (deep reinforcement learning) model [14] is adaptive to a wide range of environments and scenarios, making them potentially more flexible than rule-based systems or systems based on supervised learning. DRL aims to maximize a reward function, which can be tailored to optimize for various objectives like safety, efficiency, or ride comfort. However, while DRL agents are often trained in simulated environments, transferring learned policies to the real world (“sim-to-real transfer”) can be challenging due to differences between the simulation and reality.

Al Sallab et al., 2016 [15], uses a convolutional neural network (CNN) for feature extraction and spatial understanding. However, the action values still need to be discrete, which can be a limitation in cases requiring continuous action values. In addition, an imitation learning (IL)-based model has been proposed [16]. It tried to mimic the behavior of an expert. Dataset aggregation techniques are often used in imitation learning but the impact on safety is not considered. Since IL methods learn to imitate human actions, they might inherit human errors or sub-optimal behaviors, which is an issue for safety-critical applications.

Overall, we observed that LSTM-based models are computationally intensive, which could be a drawback for realtime applications where low latency is crucial. Also, DRL-based and IL-based autonomous driving algorithms require a large number of samples or experiences to learn effectively, which are computationally expensive and time-consuming. Hence, this study adopted a simple but effective CNN-based driving model provided by NVIDIA (Santa Clara, CA, USA), which is an end-to-end learning model for self-driving cars and also suitable for our test platform of RC cars and a CARLA simulator [17,18]. Note that the choice of the best driving algorithm is not our objective in this paper. Our goal is to identify the realtime issues of self-driving and propose a solution for them.

## 3. Environment-Driven Autonomous Driving

Autonomous driving is a mission-critical operation in the sense that on-time processing is very important for driving a car in a real-world environment. Specifically, autonomous driving is not a simple task but more like a set of tasks consisting of various jobs including image processing, machine learning, motor control, and so on. For autonomous vehicles to safely drive in complex environments, autonomous cars should ensure deadlines for sensors systems and car-controlling algorithms including machine learning modules, which are known to be very computationally intensive.

However, the deadlines and runtimes of these above modules are far more complicated in the real world. For example, the required deadlines of each algorithm in autonomous vehicles can vary in different scenarios. In the case of driving in a downtown area with a low speed limit, a slower but more accurate prediction might be required than for high-speed highways. Also, there are huge gaps between the worst and average run times of each module, especially in real-world dynamic environments. This implies that current realtime systems focusing on static and strict deadlines might not be suitable for such dynamic and fast moving autonomous driving environments.

To address this issue, we first (1) discover realtime factors hindering level 5 self-driving by examining detailed effects of these factors and then (2) propose an environment-driven solution to balance the trade-off between runtime accuracy and end-to-end delay.

### 3.1. Realtime Factors

**Figure 2:**
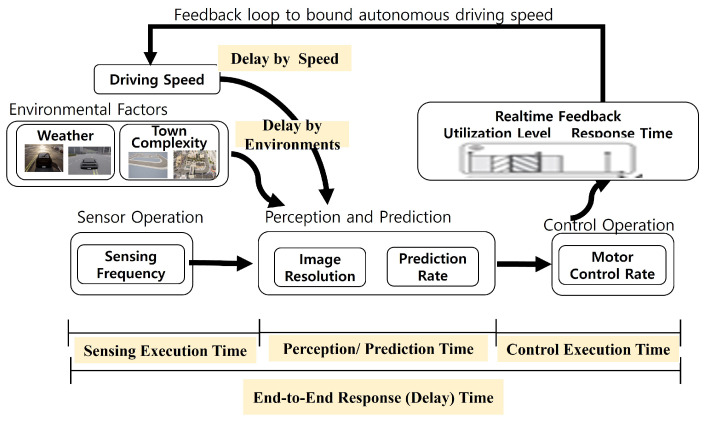
Feedback loop of the proposed approach.

We have examined internal and external factors which can affect the realtime operation of self-driving. As illustrated in Figure 1 and Figure 2, an autonomous car consists of lots of hardware and software modules, and surrounding environmental factors are also very diverse. All of these factors affect the end-to-end response delay of a driving operation. Here is a subset of realtime factors, which the proposed approach will exploit further in Section 3.2.

- Sensing frequency
  Generally, autonomous cars are equipped with many sensors including cameras, radars, LiDARs, and so on. These sensors operate at different frequencies and their sensing frequency can be set up in a dynamic way, considering driving environments.
- Image resolution
  The image sensors for autonomous vehicles are divided into two parts—one for passengers and the other for the computational algorithms that guide the vehicle. In this paper, we focus on the second case. It can be thought that higher-resolution, higher-end cameras produce ‘better’ images and ultimately lead to ‘better’ autonomous driving results. However, a high-spec camera can cause extra loads on the CPU and GPU. So, a minimum image resolution should be determined by external factors, such as weather and town complexity, and should be bounded by core utilization.
- Prediction rate
  Depending on the complexity of the algorithms, the execution times of perception and prediction operations can vary widely, and even further runtime of these operations can be affected by other factors such as image resolutions, overloaded levels of CPU/GPU, and so on. Therefore, increasing the prediction frequency for a faster response is not always possible or desirable from an end-to-end latency perspective.
- Vehicle speed
  It is trivial that a fast driving car requires higher frequencies of sensing and prediction for prompt motor control with a short deadline of end-to-end response time. To avoid missing a deadline, we might need to limit speed or raise the frequencies of sensing and prediction. Or we might need to decrease the execution time of required operations. The proposed approach in this paper balances these operation frequencies, runtime, and preemption time to bound its end-to-end response delay for safe autonomous driving.
- Weather
  It is a well-known fact that perception and sensing for autonomous driving under adverse weather conditions has been a big problem. Many studies have been proposed to solve this problem, focusing on image processing algorithms with high-quality cameras with special capabilities [19,20]. However, these complicated and high-spec cameras and sensors are not enough to enhance autonomy because these high-power functionalities might increase execution time and response delay. This paper identifies the trade-offs and relationships between image resolution and end-to-end delay time.
- Complexity of circumstances
  Depending on town complexity, such as traffic volume, pedestrian density, traffic signals, and road maps, the required perception and prediction rates and accuracy of autonomous driving systems can vary. Furthermore, these environmental external factors can result in different or even inaccurate outputs of perception and prediction operations.

### 3.2. Proposed Solution

In this section, we propose a solution to address the issues mentioned in the previous section. Traditional realtime scheduling and algorithms estimate a runtime of each module and deadlines for the operations are given by the system. In this paper, we propose a new framework by changing the directions of the process operation, i.e., the environment-driven control of autonomous cars. For safe level 5 driving, we first examine the environmental factors that we cannot control at all, and then we set up internal factors (sensing frequency, image resolution, prediction rate, car speed, and so on). By considering the trade-off between runtime accuracy and delay, we can set up its feasible deadlines and the internal parameters of the sensors and algorithms. We call the proposed framework a environment-driven harmonized approach for autonomous driving.

Specifically, we formally formulate the observation in the previous section as follows. The objective of the following formulations is to obtain the allowable speed of a target self-driving car and to identify sensing/prediction frequency and image resolution levels. To obtain these driving parameters, we need to figure out the allowable maximum end-to-end response times of the driving operation. As mentioned in Figure 1, the end-to-end response delay includes execution times of sensing, prediction, and motor control processes. Since these processes might be interrupted and preempted by other operating system processes and communications, the end-to-end response delay can be dynamically extended. Considering these dynamic realtime issues, we set up the $Delay^{Env}$ value for each environmental level. Note that, in this paper, we consider a simple camera-based autonomous driving car just like a Tesla autopilot car [6] as a target system. This work can be easily extended for other types of self-driving cars equipped with more sensing modules such as LiDARs, GPS, and so on.

The worst case end-to-end delay, $delay_{e2e}$, of an operation flow from input factors captured by sensors to a motor control via the driving algorithms shown in Figure 1, which can be obtained from the following mechanism. This mechanism was originally developed for estimating the worst case end-to-end delay of sensor networks [21,22]. We adopted this verified theory for estimating an end-to-end delay of autonomous driving operation flow. We consider the scenario that, at each operation module, there can be multiple inputs of flow to wait to be processed. In this case, the worst case time $w_{i}\left(q\right)$ to flush the first *q* inputs is calculated as follows:

(1) $$w_{i}\left(q\right)=\left\lceil \frac{q*e_{i}^{r}}{s_{i}}\right\rceil *p_{i}.$$

This equation is understood as follows: the required time for processing *q* images is $q*e_{i}^{r}$, where $s_{i}$ is an execution slot time assigned to a module $m_{i}$ for processing images. So, $\left\lceil \frac{q*e_{i}^{r}}{s_{i}^{r}}\right\rceil$ can be the required number of $s_{i}$s. Hence, w(q) to finish processing the first *q* images can be obtained by adding $w_{i}\left(q\right)$ along the processing pipeline.

**Figure 3:**
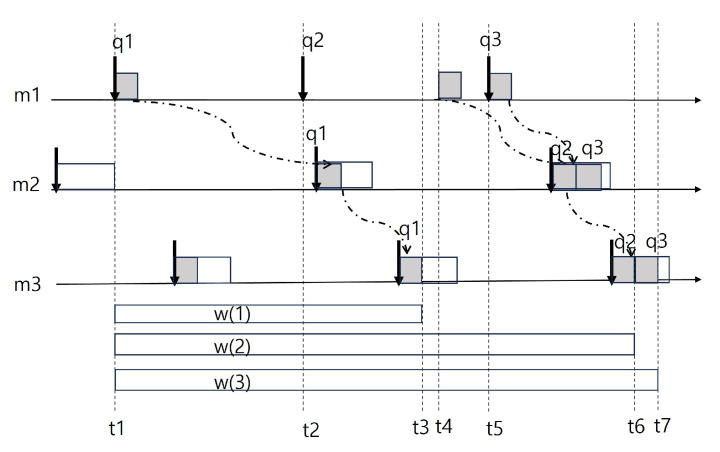
Worst case end-to-end delay.

Now, let us look at the example in Figure 3. In this example, we assume a scenario in which data are transmitted to each module, $m_{1}\to m_{2}\to m_{3}$. When $p_{i}$ is a period of a module $m_{i}$, $m_{1}$ processes the first image input $q_{1}$ at $t_{1}$, and the second one $q_{2}$ is at $t_{4}$ not at $t_{2}$, because other operations are processed. The second image is delayed until $t_{4}$. In this way, the first image is finished at $t_{3}$ while second and third ones are finished at $t_{6}$ and $t_{7}$, respectively. In that way, the *q*th image is scheduled to be generated at (q−1)∗p and hence its end-to-end delay can be calculated as follows:

(2) w(q)−(q−1)∗p.

So, the worst case end-to-end delay, $delay_{e2e}$, can be considered the largest value among all possible values of w(q)−(q−1)∗p. Note that, in this formulation, it is already proved [21] that the maximum value *Q* exists with q=1,2,⋯,Q, where *Q* is the first integer that satisfies w(q)≤Q∗p. The worst case delay, $delay_{e2e}$, can be calculated as follows:

(3) $$delay_{e2e}=max_{q=1,2,\cdots Q}w\left(q\right)-\left(q-1\right)*p.$$

Now, using the above formulas, we design an admission control module with the following parameter constraints. These constraints allow us to control internal factors such as image resolution and detection period to determine whether a given parameter is acceptable or not for supporting end-to-end deadlines in the current environmental scenario, while maintaining the accuracy of autonomous driving. For example, maintaining a high-enough autonomy for self-driving on rainy days may require higher resolution images, which can increase processing times and end-to-end delays. But at the same time, driving in the rain may require a shorter period of operation for quick reaction, which can reduce end-to-end delays. Additionally, driving on rainy days requires a longer marginal delay considering the braking distance. Overall, in a given environment, these controllable internal factors and driving speed must be considered in a harmonized way. To address these issues, the following constraints are developed:

(4) $$\begin{array}{ccc} \; & \sum_{i\in T}\frac{e_{i}^{r}}{p_{i}}\leq U_{bound}^{max}\\ \; & r_{env}\leq r\leq r_{max} & \\ \; & P_{i}^{min}\leq p_{i}\leq P_{i}^{speed_{k}} & ,i\in T\\ \; & e_{i}^{r_{env}}\leq e_{i}^{r}\leq e_{i}^{r_{max}} & ,i\in T\\ \; & delay_{e2e}+Delay^{speed_{k}}\leq Delay^{ENV}\\ . \end{array}$$

Based on these constraints, we can also achieve various objectives considering our system design goals. For example, we can minimize total utilization for potential availability or we can maximize image resolution for higher accuracy.

Note that the above constraints in Equation (4) are parameter filters, which cause polynomial computation complexity. However, the additional optimizing process in Equation (5) with these constraints is converted to an ILP problem. An ILP-based method used in the proposed admission control module is known to be NP-complete [23,24]. However, it is also proved that it can be solved by a pseudo-polynomial algorithm [25,26] with some given minimum and maximum values. The proposed method uses this heuristic ILP solver [27] to reduce the search space.

(5) $$\begin{array}{ccc} \; & \mathrm{minimize}U_{bound}^{T}=\sum_{i\in T}\frac{e_{i}^{r}}{p_{i}}, & \\ \; & \;\mathrm{or}\;\\ \; & \mathrm{maximize}r, & \\ . \end{array}$$

The main notations used in Equations (4) are presented in Table 1. Here, we introduced three types of delay notations—$delay_{e2e}$, $Delay^{speed_{k}}$, and $Delay^{ENV}$. $delay_{e2e}$ is the end-to-end response time of the current internal parameters, while $Delay^{ENV}$ is a desired maximum response delay to support autonomous driving for given environmental factors. $Delay^{speed_{k}}$ is a marginal delay to accommodate driving speed. The braking distance, also called the stopping distance, is the distance a vehicle covers from the time of the full application of its brakes until it has stopped moving. Considering the breaking distance, we set up additional delay factors for each driving speed level [28].

In the above equations, the proposed approach keeps the utilization of ECU below its utilization bound [29] so that it can handle a critical and urgent situation (such as the sudden appearance of pedestrian, cars, or anything dangerous) quickly with the highest priority without scheduling problems. So, depending on the current core utilization, the execution time and period (=rate) of a task can be bounded to some extent. Also, $speed_{k}$ is determined as the desired maximum speed level of a car and is defined as 1 (=low), 2 (=medium), 3 (=high), or 4 (=very high) (these levels can be easily extended to accommodate a real-world driving scenario. Also, there can be more internal factors affecting execution time. For simplicity of explanation, we here examine image resolution and sensing frequency). Periods (i.e., a reverse of frequencies) of sensing, prediction, and motor control operations are denoted as $p_{i}$. The execution time of each process, $e_{i}^{r}$, is also bounded depending on a desired image resolution to satisfy desired accuracy, which is represented as $r_{env}$. Environmental factors affect the required image resolution, which in turn affects the expected execution time of each process. Note that execution times of operations can be further delayed by preemption from OS-related high priority processes. In this study, VxWorks [30,31] is adopted as a realtime operating system in the developed RC car. VxWorks is one of the main embedded realtime operating systems (RTOSs). In Vxworks, periodic tasks such as sensing operations are scheduled by round-robin scheduling while non-periodic tasks are scheduled by static priority-base preemptive scheduling. To reflect Vxwork scheduling-related additional delay, execution times calculated in this study implicitly include the potential preemption delay as well.

The procedure of the proposed algorithm is well illustrated in Algorithm 1. In the following, we explain the details of each function.
**Algorithm 1:**Environment-driven harmonized approach.1:**procedure**Proposed Algorithm2:    Collect Env (=Environmental factors)3:    Setup end-to-end delay based on Env (= $Delay^{Env}$)4:    Run optimizing_operation()5:    Set internal factors, $speed_{k}$, image resolution, sensing/prediction frequency6:    **while** Driving **do**7:        Monitor environmental changes and autonomy levels8:        **if** environments change **then** continue to line 2;9:        **end if**10:        **if** autonomy≤threshold **then** adjust internal factors.11:        **end if**12:    **end while**13:**end procedure**

- <line 2>: Collect environmental factors such as weather, town complexity, road conditions, and so on.
- <line 3>: As a proactive operation, we first set up a desired end-to-end response (delay) time, $Delay^{Env}$ considering the collected environmental information. Specifically, $Delay^{Env}$ is determined by reflecting the effects of bad weather and crowded complex driving circumstances in this paper.
- <line 4>: Run optimizing_operation() described in Equation (4).
- <line 5>: Using output values of the above optimizing operation, set up maximum allowable speed, image resolution, sensing, and prediction frequencies. Note that optimizing_operation calculates multiple values or bounds for each internal factor. We pick the median value at first and tune them based on driving feedback.
- <line 7–9>: During driving, a self driving car keeps gathering information about environmental factors. When it detects meaningful changes, repeat the procedures from line 2.
- <line 10>: If the self-driving level has fallen below a threshold, internal factors are adjusted. We configure the desired end-to-end delay from sensors to the controlled motor to support safe autonomous driving.

## 4. Evaluation

### 4.1. Experiment Setup

**Figure 4:**
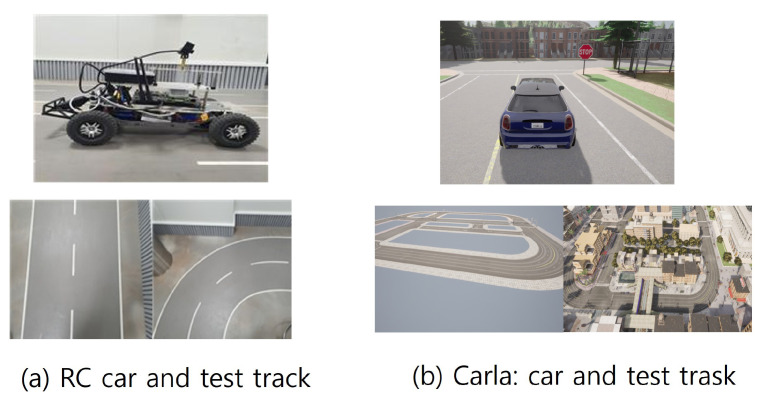
Experimental setup: (**a**) RC car and (**b**) CARLA simulator.

To evaluate the proposed solution, we conducted experiments with a real RC car testbed, which we built, and a CALRA simulator [32] as shown in Figure 4. In this paper, we use the following two metrics to evaluate the accuracy of autonomous driving.

- Comparing Ground Truth and Predicted Value: To evaluate self-driving cars, we compared the true values and the predicted values. For example, we have the ground truth values of the steering angle when we perform manual driving to collect data. The steering value for each snapshot image is considered the ground truth value. Then, we use our model to predict the steering value for each image. With the comparison results, we computed MAEs (Mean Absolute Errors), which is the most commonly used method for evaluating self-driving cars.
- Autonomy: Autonomy is often used to refer to the ability of individuals or organizations to make their own choices without interference from others. We use this concept to evaluate autonomous cars. Specifically, in the case of self-driving cars, autonomy can represent how much the cars drive by itself. Through this metric, we can find out how much time the car drives by itself for without a human driver. Formally, we formulate autonomy as the following Equation (6). In this equation, intervention_time is defined as the average time taken for each human intervention operation. In this study, we assume 6 s, which can be adjusted any time based on the target system and road conditions.
  (6) $$autonomy=\frac{1-(number\_of\_interventions)*intervention\_time}{elapsed\_time(s)}*100.$$

Our experiments use a CNN-based autonomous driving algorithm provided by NVIDIA [17]. Note that we are intending to verify the effects of realtime factors in the proposed solution rather than evaluating the performance of an algorithm itself. The basic training parameters of the machine learning model contained in the proposed approach are shown in Table 2.

### 4.2. Experimental Results

In this paper, the objective of the proposed system is to bound an end-to-end response delay of an autonomous driving operation by setting up internal factors considering the environments, which are not controllable factors. Through extensive experiments varying several internal and external environment factors, we observed several interesting points. Specifically, in Section 4.2.1 and Section 4.2.2, we present a summary of our observations related to the accuracy and autonomy results of self-driving tests for each external and internal factor. These results are then used to set realtime parameter values in Section 4.2.3 in turn.

The main focus of the proposal is not to develop the autonomous driving algorithm itself. Instead, we provide a framework for setting realtime relevant parameters to limit the end-to-end delay for a given autonomous driving algorithm. Therefore, we did not attempt to improve or modify NVIDIA’s ML algorithm for better driving results in our evaluation. Instead, we observed how environmental and internal controllable factors influenced the results and designed an admission control module to setup realtime-relevant parameters.

#### 4.2.1. Effects of External Factors

**Figure 5:**
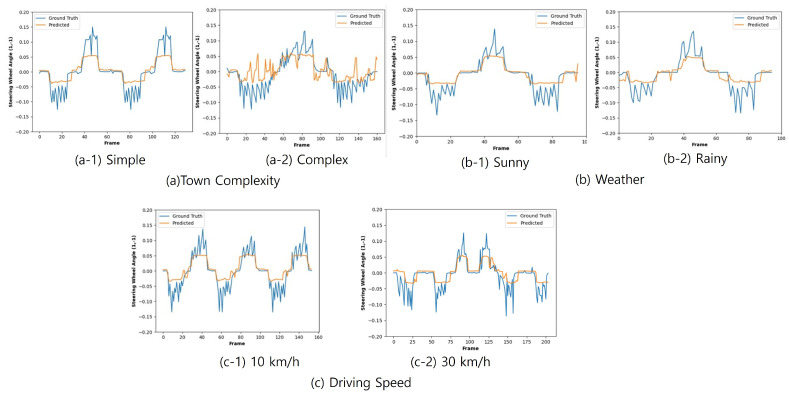
Comparison results: ground truth vs. prediction: (**a**) town complexity, (**b**) weather, and (**c**) speed.

We first examined the effects of environmental factors such as weather and complexity on driving circumstances as well as driving speed. In this analysis, as a metric, we compared the prediction outputs with the corresponding ground truth steering values. Figure 5a,b show the accuracy effects of these environmental factors, which we cannot control. Figure 5c shows the comparison results with two different car speeds—10 km/h and 30 km/h. Table 3 shows the MAE values for each factor. This analysis shows that accuracy is degraded as weather and driving circumstances become bad. In contrast, the given algorithm seems to perform similarly regardless of driving speed. This observation can be explained by the fact that the above comparison experiments represent the steering prediction accuracy for each picture frame. Hence, driving speed does not affect these accuracy results as much while weather and driving circumstances can degrade accuracy because these external factors can make perception processes such as lane detection worse. However, as we mentioned before, this MAE-based comparison metric just shows a snapshot result of each instance, not accumulated, and sequential driving results. Hence, we examined the effects of car speed using a different metric, autonomy, as we explained in the previous Section 4.1.

#### 4.2.2. Effects of Internal Factors

**Figure 6:**
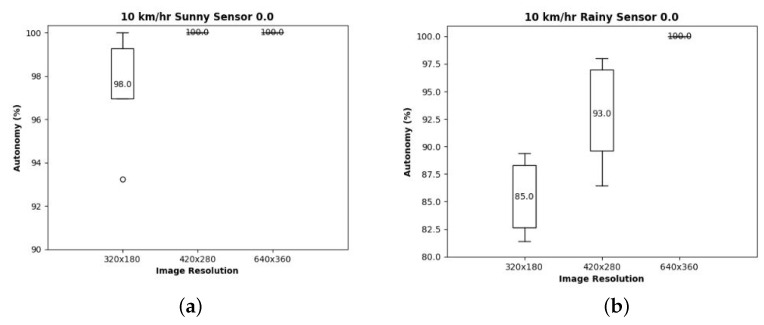
Autonomy for each image resolution: (**a**) sunny and (**b**) rainy weather.

**Figure 7:**
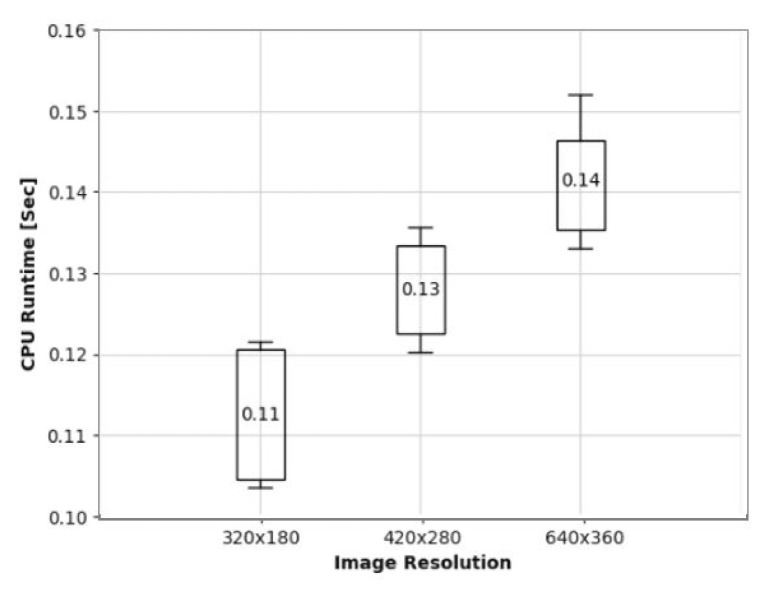
Execution time for each image resolution.

We also examined the effects of internal parameters or factors of operations, which we can control in contrast to environmental factors; first, to see the effects of image resolutions on autonomous driving, With these experiments, we tried to answer the question of whether higher resolution is always better or not. Specifically, we conducted test driving with three different image resolution cameras—320 × 180, 420 × 280, and 640 × 350. As shown in Figure 6, on a sunny day, autonomy is almost 100% regardless of image resolutions, while on a rainy day, low resolution images result in only 80% autonomy. To examine the effects of image resolution on runtime, we also presented the CPU execution time results in Figure 7, which shows almost 30% more execution time usage with a higher resolution. Based on these above observations, there is definitely a trade-off between image resolution and execution delay.

**Figure 8:**
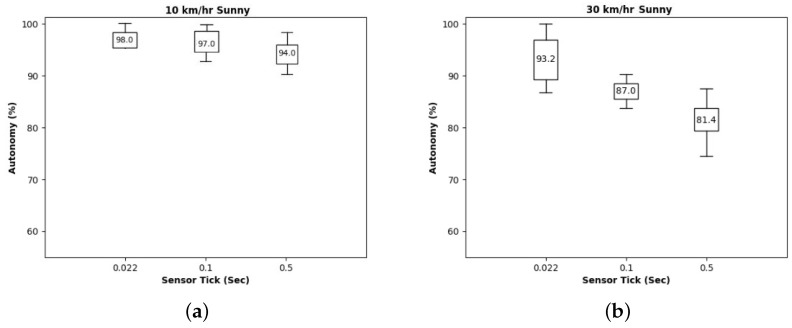
Autonomy for each sensing frequency: (**a**) 10 km/h and (**b**) 30 km/h speed.

Now, we examine the effects of sensing frequency with varying intervals between camera sensor operations. In Figure 8, we showed three results with intervals of 0.022 s (which is the minimum in our testbed), 0.1 s, and 0.5 s. Two graphs show the autonomy results at 10 km/h and 30 km/h, respectively. We observe that fast driving requires more frequent sensing, which requires a short deadline of end-to-end response time. In the case of slow driving, sensing frequency does not much affect autonomy while a lower frequency degrades autonomy a lot with a fast moving car. Of course, raising sensing frequency also increases the CPU’s utilization of the system, which might bound maximum frequency. This implies that we need to adjust sensing frequency based on speed when considering CPU execution time as well.

#### 4.2.3. End-to-End Delay Analysis

In the previous section, we examined accuracy and autonomy for each external environmental factor and internal factor. We use these analyses to determine whether a given parameter is acceptable or not for supporting end-to-end deadlines in the current environmental scenario while maintaining the accuracy of autonomous driving. In particular, several parameter values in Equation (4) can be set up based on these results. For example, in our test scenario, we set up $r_{env}$, $P_{i}^{speed_{k}}$, $Delay^{ENV}$ as follows to maintain accuracy and autonomy above a certain threshold. Note that threshold values are dependent on target systems and platforms, so these values can be customized further. In this paper, we set up the autonomy and accuracy (i.e., MAE) thresholds as 90% and 0.025, respectively. Also, $r_{min}$ and $P_{i}^{min}$ are determined by the system hardware configuration. In our experiments, these values are set up as 320 × 180 and 0.022 s, respectively.

**Figure 9:**
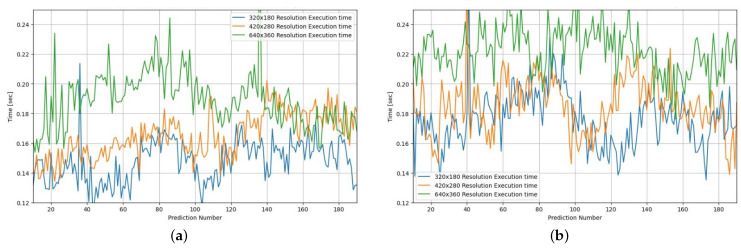
End-to-end delay time: (**a**) sensing frequency 0.022/s and (**b**) sensing frequency 0.1/s for each image resolution frequency.

Figure 9 shows sample experimental end-to-end delay results in detail. Specifically, it presented the end-to-end delay times with varying sensing frequencies, 0.022/s and 0.1/s for three different image resolutions on a rainy day at a 30 km/h speed. Based on the realtime admission control parameters shown in Table 4, the sensing period should be smaller than 0.022 and image resolution should be larger than 420 × 180. So, the bottom line in the right graph of Figure 9b represents unacceptable parameter combinations in a scenario of 30 km/h speed self-driving on a rainy day because its image resolution is lower than expected and so it might not generate accurate self-driving direction and so cannot provide a high level of autonomy. On the other hand, in the case of the top line in Figure 9b, the corresponding combination of parameters is not accepted either because its end-to-end delay does not satisfy the constraints in Equation (4). So, we need to lower the image resolution to reduce the execution time, or need to increase the sensing frequency.

Table 5 shows four samples of the parameter combinations we got from the proposed optimizer for various environments and three samples which are not accepted by the optimizer. The fourth and fifth samples are not acceptable because their expected autonomy values are below 90%. Note that the last sample in the table shows that its 70% end-to-end delay is about 0.32 s, which can be considered as highly risky to be accepted. This is because in rainy weather the required end-to-end deadline, $Delay^{ENV}$, would be less than 0.4 s (i.e., safe response time in the normal scenario) and also a marginal delay with the breaking distance value is 0.1 s in this case. Hence, a 0.32 s delay would be considered risky from a conservative point of view.

Overall, we observed that external and internal factors are heavily related to each other and changing these factors might increase autonomy at the cost of execution time delay and total CPU utilization consumption. Even in such a simplified RC car and a CALRA simulator model, these factors are tightly coupled and so harmonizing these factors to satisfy the end-to-end response delay is not straightforward. The proposed admission control module with parameter constraints in Equation (4) allows us to control internal factors such as the image resolution and detection period to determine whether a given parameter is acceptable or not for supporting end-to-end deadlines in the current environmental scenario while maintaining the accuracy of autonomous driving.

## 5. Conclusions

The autonomous driving industry has developed and is attempting to achieve level 5 self-driving in the real world. This paper tackles the “last mile problem” in the journey towards level 5 self-driving, Autonomous driving is a mission-critical operation in the sense that on-time processing is very important for driving a car in a real-world environment. So far, most researchers have focused on improving the accuracy of perception and prediction methods and models. In contrast, there have been fewer studies identifying the relationship and trade-off between runtime-accuracy and the end-to-end delay of autonomous driving operations. Since autonomous driving operations consist of several different stages of a process, it is very important to complete the flow of these processes within some time constraints. Specifically, autonomous driving is not a simple task but more like a set of tasks consisting of various jobs including image processing, machine learning, motor control, and so on. For autonomous vehicles to safely drive in complex environments, autonomous cars should ensure the end-to-end delay deadlines of sensor systems and car-controlling algorithms, including machine learning modules, which are known to be very computationally intensive. To address this issue, we proposed a new framework, i.e., an environment-driven approach for autonomous cars. We investigated and discovered realtime factors that hinder level 5 autonomous driving. We identified environmental and internal factors with extensive experiments. We examined and detailed the effects of these factors and measured the relationships and trade-offs among these factors based on experimental results using both RC cars that we built and CARLA simulators. Then, we proposed an environment-driven solution to address these issues.

With the proposed solution, we properly set up internal factors (sensing frequency, image resolution, prediction rate, car speed, and so on) by considering the trade-off between runtime-accuracy and delay. The proposed approach was validated using both an RC car and a simulator. The results showed that the realtime-relevant parameters of the proposed optimizer bounded the end-to-end delay within the desired deadline and maintained high accuracy and an autonomy level of self-driving.

In future work, the realtime relevant parameters presented in this paper can be further extended to include more sensors such as LiDAR, radar, and GPS, with more driving-related tasks such as map generation and localization, etc. Additionally, the proposed access control module can be customized to suit various realtime scheduling methodologies.In this paper, we attempted to solve realtime-related problems and showed the feasibility of a solution that reconciles controllable internal and uncontrolled environmental factors to keep accuracy high and to limit end-to-end delay. We believe that this work will serve as a guide to solving the last mile problem of level 5 autonomous driving.

## Figures and Tables

**Table 1:** Notations in the proposed utilization-driven approach.

Notations	Definitions
$t_{s},t_{p},t_{c}$	sensing task, prediction task, and motor control task, respectively
T	target task set (e.g., =$\left\{t_{s},t_{p},t_{c}\right\}$)
$e_{i}^{r}$	execution time of task $t_{i}$ with a image resolution value *r*
$r,r_{max},r_{env}$	current, maximum, and required image resolution for environmental factors
$p_{i}$, $p_{min}$	current and minimum period of task $t_{i}$
$U_{bound}^{max}$, $U_{bound}^{T}$	maximum and current utilization bound of task set T
$speed_{k}$	the speed level *k* = {1 (=low), 2 (=medium), 3 (=high), 4 (=very high) }
$P_{i}^{speed_{k}}$	required maximum period of task $t_{i}$ at the car driving speed level, $speed_{k}$
$delay_{e2e}$	the end-to-end delay of the current auto driving car
$Delay^{speed_{k}}$	desired marginal delay to accommodate driving speed [28]
$Delay^{ENV}$	desired end-to-end delay considering environments

**Table 2:** Machine learning training parameters.

Parameter	Value
Number of Image Channels	3
Batch Size	32
Width Crop	0
Height Crop	90
Number of Images	4370
Number of Epochs	100
Learning Rate	0.001
Optimizer	Adam

**Table 3:** MAE (Mean Absolute Error) of comparing predicted outputs with ground truth values.

	Weather		Driving Circumstance		Speed	
	**Rainy**	**Sunny**	**Simple**	**Complex**	**10 km/h**	**30 km/h**
MAE	0.01993	0.02434	0.021	0.031	0.022	0.0235

**Table 4:** Sample realtime parameters in our test scenario of a simple town.

	Sunny Weather		Rainy Weather	
	**10 km/h**	**30 km/h**	**10 km/h**	**30 km/h**
$r_{env}$	320 × 180	320 × 180	420 × 280	420 × 280
$P_{i}^{speed_{k}}$	0.5	0.022	0.1	0.022
$Delay^{speed_{k}}$	0.1	0.15	0.13	0.18
$Delay^{ENV}$	0.5	0.5	0.4	0.4

**Table 5:** End-to-end response time for each input factor.

Environment	Input Factors			Driving Results			
**Weather**	**Speed**	**Resolution**	**Sensing Period**	**Autonomy**	**End-to-End Delay (s)**		
					**30%**	**70%**	**Avg**
Sunny	10	320 × 180	0.022	98%	0.13	0.18	0.15
Sunny	30	420 × 280	0.1	97%	0.15	0.21	0.16
Sunny	10	420 × 280	0.5	94%	0.16	0.23	0.21
Rainy	10	420 × 280	0.022	93%	0.14	0.21	0.17
Rainy	30	640 × 360	0.5	89%	0.13	0.26	0.18
Rainy	10	320 × 180	0.022	85%	0.13	0.18	0.15
Rainy	30	640 × 360	0.1	94%	0.15	0.32	0.21

## Data Availability

Data are contained within the article.

## Abbreviations

The following abbreviations are used in this manuscript:

ML	Machine Learning
LSTM	Long Short-Term Memory
ROS	Robot Operating System
DRL	Deep Reinforcement Learning
CNN	Convolutional Neural Network
RTOS	realtime operating system
IL	Imitation Learning
MAE	Mean Absolute Errors
